# HTA community perspectives on the use of patient preference information: lessons learned from a survey with members of HTA bodies

**DOI:** 10.1017/S0266462324000138

**Published:** 2024-03-05

**Authors:** Mickael Hiligsmann, Barry Liden, Charlotte Beaudart, Evi Germeni, Alissa Hanna, Maya Joshi, Catherine P. Koola, Barry Stein, Mandy Tonkinson, Deborah Marshall, Simon Fifer

**Affiliations:** 1 Department of Health Services Research, CAPHRI Care and Public Health Research Institute, Maastricht University, Maastricht, The Netherlands; 2 Public Policy, USC Schaeffer Center for Health Policy & Economics, Los Angeles, CA, USA; 3 NARILIS (NAmur Research Institute for LIfe Sciences), University of Namur, Namur, Belgium; 4 Health Economics and Health Technology Assessment (HEHTA), School of Health and Wellbeing, University of Glasgow, Glasgow, UK; 5 Patient Engagement, Edwards Lifesciences, Irvine, CA, USA; 6 Community and Patient Preference Research (CaPPRe), Sydney, NSW, Australia; 7 Institute for Clinical and Economic Review (ICER), Boston, MA, USA; 8 Colorectal Cancer Canada (CCC), Montreal, QC, Canada; 9 Public Involvement Programme, National Institute for Health and Care Excellence, Manchester, UK; 10 Department of Community Health Sciences, Cumming School of Medicine, University of Calgary, Calgary, AB, Canada; 11 Department of Medicine, Cumming School of Medicine, University of Calgary, Calgary, AB, Canada

**Keywords:** assessment, decision-making, health technology, HTA, patient involvement, patient preferences, patient outcomes

## Abstract

This research sought to assess whether and how patient preference (PP) data are currently used within health technology assessment (HTA) bodies and affiliated organizations involved in technology/drug appraisals and assessments. An exploratory survey was developed by the PP Project Subcommittee of the HTA International Patient and Citizen Involvement Interest Group to gain insight into the use, impact, and role of PP data in HTA, as well as the perceived barriers to its incorporation. Forty members of HTA bodies and affiliated organizations from twelve countries completed the online survey. PP data were reported to be formally considered as part of the HTA evidence review process by 82.5 percent of the respondents, while 39.4 percent reported that most of the appraisals and assessments within their organization in the past year had submitted PP data. The leading reason for why PP data were not submitted in most assessments was time/resource constraints followed by lack of clarity on PP data impact. Participants reported that PP data had a moderate level of influence on the deliberative process and outcome of the decision, but a higher level of influence on the decision’s quality. Most (81.8 percent) felt patient advocacy groups should be primarily responsible for generating and submitting this type of evidence. Insights from the survey confirm the use of PP data in HTA but reveal barriers to its broader and more meaningful integration. Encouragingly, participants believe obstacles can be overcome, paving the way for a second phase of research involving in-depth collaborative workshops with HTA representatives.

## Introduction

Interest in patient preference data to aid in health technology assessment (HTA) and inform payer decisions has drastically increased (1;2). Defined by the US Food and Drug Administration as “qualitative or quantitative assessments of the relative desirability or acceptability to patients of specified alternatives or choices among outcomes or other attributes that differ among alternative health interventions,” patient preference information (or data) therefore differs from other forms of patient involvement, such as patient reported outcomes aiming to gather information on a patient’s disease or treatment experience. In particular, highly sophisticated, quantitative preference elicitation and analysis methods designed to maximize scientific certainty and minimize potential for bias (such as discrete-choice experiments (DCEs)) are increasingly being used to quantify patient preferences through statistical analysis (3). Frameworks for patient preference information have been recently developed, including those from the Medical Device Innovation Consortium and the Innovative Medicines Initiative-PREFER project. Several HTA agencies have furthermore now begun including evidence from such studies in their guidelines (1). Several initiatives are also being undertaken to expand the usefulness and impact of patient preference data in healthcare decision-making (4;5).

While enthusiasm for the potential value of patient preference data is growing, information about the current use of such data in HTA is lacking. The Patient Preference Project Subcommittee (PPPS) of the HTA International (HTAi)’s Patient and Citizen Involvement Interest Group (PCIG) has therefore undertaken research to improve understanding about whether and how patient preference data are currently used within HTA. As a first step, this article reports the results of a survey conducted among members of HTA bodies and affiliated organizations involved in technology/drug appraisals and assessments to determine their views on incorporating patient preference research into HTA. This research aims to set the scene for future patient preference evidence to support HTA decision-making in more robust and sophisticated ways and to align with the expectations of members of HTA bodies.

## Methods

The survey was developed and administered by the HTAi’s PCIG PPPS to solicit feedback on the use of patient preference data within HTA organizations.

### Survey Development and Content

The survey was developed, reviewed, and approved by the HTAi PCIG PPPS members, including patients, drug and device industry, health technology assessors, policy makers, and (academic and private) researchers with expertise in patient preference research. These representatives came from Europe, North America, Australia, and Asia. The first section of the survey consisted of a series of background questions including capturing information about the participant’s country of residence, organization, and familiarity with preference elicitation approaches. Participants were then asked to share their perspectives on the use and impact of patient preference data in HTA, and the role and responsibilities of patient preference data in HTA. Perceived barriers to incorporating patient preference data in HTA were also requested. The full survey questionnaire is available upon request from the corresponding author and was executed using the online platform Confirmit.

### Survey Population and Recruitment

Members of HTA bodies and affiliated organizations who are involved in technology/drug appraisals and assessments were invited to participate. Members of the PCIG working group distributed invitations to their global HTA contact networks, HTAi shared and promoted the survey among its members, and contacts were encouraged to share the survey with others in their organizations or other contacts of theirs. PCIG working group members posted recruitment information on their personal social media profiles (i.e., LinkedIn, Twitter), and the HTAi PCIG shared information through their newsletter and posted it on the official HTAi PCIG webpage.

Data collection took place during November and December 2021. All responses were treated as confidential and participants could choose to remain anonymous; however, email addresses were collected for those who wished to be notified about the outcomes of the study. These were kept separate from the data file.

No ethics approval was deemed necessary due to the negligible risk associated with this non-interventional survey. Participants were recruited through professional networks, and no sensitive information was collected. Under the Code of Federal Regulations, this research falls within Exemption 45 CFR 46.104(d)(2) for Educational Tests, Surveys, Interviews, or Observation of Public Behaviours (https://www.hhs.gov/ohrp/regulations-and-policy/decision-charts-2018/index.html#c2).

### Analysis

Participants who completed the survey in full were included for analysis. Descriptive statistics (frequency, number, median) were carried out with the software SPSS. Open-text questions were manually coded by one researcher. Classification and naming of barriers were undertaken by two researchers.

## Results

In total, 122 people accessed the survey, of which 19 were screened out due to ineligibility and 63 were incomplete. A total of 40 participants from 12 countries fully completed the survey and were included in the analysis. Most of them were from Europe (51 percent), and North America (35 percent). In total, 15 percent of participants reported working for a university, while others were from different national organizations (see Supplementary Appendix Table 1).

### Familiarity with Preference Elicitation Approaches

Most participants were at least somewhat familiar or very familiar with all five of the proposed preference methods (Table 1). Half of the sample was somewhat or very familiar with all five methods, and none of the participants claimed to be unfamiliar with all five methods presented. Qualitative methods had the highest level of familiarity among participants (97.5 percent of the sample reported to be somewhat/very familiar with this approach), while the lowest level of familiarity was attributed to the indifference method (62.5 percent somewhat/very familiar).

**Table 1.** Familiarity with preference elicitation approaches (*n* = 40)

	Number (freq.)
Familiarity with discrete choice-based methods (e.g., DCEs, conjoint)	
Very familiar	11 (27.5%)
Somewhat familiar	18 (45%)
Not familiar	5 (12.5%)
Do not know	6 (15%)
Familiarity with ranking methods (e.g., best-worst scaling)	
Very familiar	8 (20%)
Somewhat familiar	23 (57.3%)
Not familiar	4 (10%)
Do not know	5 (12.5%)
Familiarity with indifference methods (e.g., standard gamble, time trade-off)	
Very familiar	12 (30%)
Somewhat familiar	13 (32.5%)
Not familiar	7 (17.5%)
Do not know	8 (20%)
Familiarity with rating methods (e.g., constant sum, visual analogue scale)	
Very familiar	13 (32.5%)
Somewhat familiar	15 (37.5%)
Not familiar	5 (12.5%)
Do not know	7 (17.5%)
Familiarity with qualitative methods (e.g., patient stories/case studies/patient hearings)	
Very familiar	31 (77.5%)
Somewhat familiar	8 (20.0%)
Not familiar	1 (2.5%)
Do not know	0 (0%)
Very or somewhat familiar with all the five methods (frequency of those who selected “Very familiar” or “Somewhat familiar” for all five methods)	20 (50%)
Not familiar/do not know all of the five methods	0 (0%)

### Use and Impact of Patient Preference Data in HTA

Patient preference data were reported to be formally considered as part of the evidence review process for HTA by 33 (82.5 percent) of the participants (Table 2). Among the seven participants who indicated that their organization does not use patient preference data, two of them simply reported that they were not familiar with these methods. One participant from the United Kingdom reported that their organization considers these methods as “soft” evidence and therefore does not use them, whereas another participant from Poland reported that their organization is currently looking for a way to include the patient voice in their decision-making process. The remaining three did not give an explicit reason as to why their organization does not use patient preference data in HTA. Interestingly, four participants reported that they anticipate changes within their organization as patient preference data may be incorporated into the evidence review process in the next few years.

**Table 2.** Use and impact of patient preference data in HTA within organizations

	Number (freq.)
Use of patient preference data in the organization (*n* = 40)	
Yes	33 (82.5%)
No	7 (17.5%)
Proportion of technology/drug appraisals and assessments in the organization that included patient preference data in last 12 months (*n* = 33)	
Most	13 (39.4%)
Some	13 (39.4%)
None	0 (0%)
Do not know	7 (21.2%)
Presence of guidelines in the organization (*n* = 33)	
Yes	15 (45.4%)
No	11 (33.3%)
Other	6 (18.2%)
Not know	1 (3%)

Of the 33 participants who reported that their organization uses patient preference data in decision-making, 13 (39.4 percent) reported that most of the technology/drugs appraisals and assessments in the past 12 months had submitted patient preference data, while 13 others (39.4 percent) reported that only some of the appraisals/assessments had patient preference data. No participant reported that none of their appraisals/assessments included patient preference data, while seven other participants could not answer this question.

Fifteen participants (45.4 percent) of those who reported using preference information in their organization said that guidelines exist in their organization in relation to the use of patient preference data, whereas eleven (33.3 percent) reported that such guidelines do not exist. Six participants (18.2 percent) did not answer yes or no to the availability of a guideline in their organization and provided additional nuanced information. For example, in Canada, two participants reported that patient preferences are not submitted to the HTA process, but that they are directly solicited, and two other participants reported (i) that they have internal procedures that do not qualify as “guidelines” per se and (ii) that they are currently reviewing existing guidelines. One participant in Taiwan reported that they follow consensus but not written guidelines, and a final participant from the United Kingdom reported that their organization has general guidelines for estimating preference-based utilities but are relatively non-prescriptive to allow some flexibility.

### Role and Responsibilities of Patient Preference Data in HTA

Most participants agreed with the prespecified roles of patient preferences in HTA, with the most frequently chosen role being “supplementary information to assess the relative value of outcomes that are important to patients”. Participants also felt that patient advocacy groups should be primarily responsible for generating and submitting patient preference data to HTA (81.8 percent), followed by academics/universities (72.7 percent), HTA organizations/affiliates (66.7 percent), and industry (51.5 percent) (Table 3).

**Table 3.** Role and responsibilities of patient preference data in HTA (*n* = 33)

	Yes Number (freq.)
*Role of patient preference data in HTA*	
Supplementary information to assess the relative value of outcomes that are important to patients	27 (81.8%)
Supplementary information on additional outcomes that are not measured in other evidence	23 (69.7%)
Complementary information to other evidence	23 (69.7%)
Supplementary information on outcomes that are measured in other evidence	22 (66.7%)
Other	4 (12.1%)
*Responsibilities for submitting patient preference data for HTA*	
Patient advocacy groups	27 (81.8%)
Academics/universities	24 (72.7%)
HTA organizations/affiliates	22 (66.7%)
Industry (Medical device and pharmaceutical industry)	17 (51.5%)
Private research organizations	10 (30.3)
Other	4 (12.1%)
Do not know	0 (0%)

### Barriers to Patient Preferences Information in HTA

**Figure 1.**
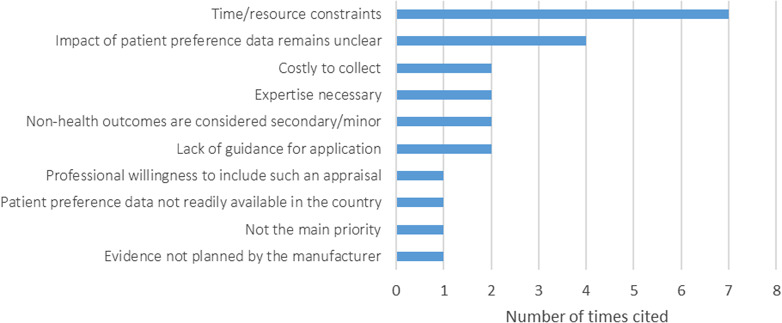
Main barriers for not submitting patient preference data in appraisals/assessments (*n* = 20).

A total of 20 barriers responsible for why patient preference data were not submitted in most assessments/appraisals were reported by participants. These barriers were then further classified into 10 main categories. The most often reported reason for why patient preference data are not submitted was the constraints of time, resources, and money (e.g., quotes from the participants: “*limited time and resources of both the patient umbrella organization and the HTA agencies*,” “*involving patients requires planning, time and budget, which are not always available*”). Four participants also indicated that the impact of collecting patient preference data remains unclear (e.g., *participant quotes: “Despite the many mechanisms that allow patients to be involved in HTA and the reimbursement process, the impact of such decision-making remains unclear*,” “*It may be that patient preferences are only a minor driver in demonstrating the value proposition of the new medicine, and therefore the company hasn’t focused its attention on gathering them*”). Examples of other barriers mentioned include the following: such assessments require expertise, the reluctance of professionals to include such an assessment, the priorities of the organization or the fact that these results are considered secondary, and so forth (Figure 1).

Among the participants, 70 percent believe that these barriers can be overcome in the next 2–3 years within their organizations. Sample quotes from participants help to illustrate this: “[these barriers can be overcome if] p*rofessionals have access to more education on the topic*,” “*If more studies focusing on patient preferences can be conducted in the region, then the barriers can be overcome*,” “*The strategic plan pushes for more patient involvement, including co-design activities with patients.*”

Despite these encouraging quotes, other participants reported less positive points of view: *“Despite being important, it is unlikely that these assessments may be one of the most important outcomes in the near future,”* “*I am not confident that there will be a sufficient change in global HTA methods that would incentivize an increased focus and investment in eliciting patient preferences in the next three years*,” “*Change takes time and more work is needed to promote this type of evidence as valid and rigorous.*”

### Influence of Patient Preference Data on Decision-Making Aspects within the Organization

Participants were asked to rate the level of influence patient preference data have on different aspects of decision-making within their organization, on a scale from 1 (“no influence at all”) to 10 (“strong influence”). In general, participants reported patient preference data have a moderate level of influence on the *process of deliberation* (median = 6) and the *outcome of the decision* (median = 6), but a higher level of influence on the *quality of the decision (*median = 7) (Table 4).

**Table 4.** Influence of patient preference data on decision-making aspects within the organization (*n* = 33)

	Median (IQR)
Process of deliberating (0 (“no influence at all”)–10 (“strong influence”)	6 (5–8)
Quality of the decision (0–10)	7 (5–8)
Outcome of the decision (0–10)	6 (5–8)

After rating all three aspects of decision-making, participants were asked to explain the reason for their answers in an open-text question. Data from this question were coded thematically, and revealed that patient preference data are considered as a part of the evidence (*N* = 9) but are often secondary to other evidence presented (*N* = 4) as is displayed in the following quotes: *“As a stand-alone, it is not as influential as other pieces of evidence, but it remains of importance,” “Although patient preference data enhance the quality of the committee’s discussion, it rarely has a direct influence on the decision (weight is still placed on published evidence from studies of effectiveness and safety).* *”*

However, many participants still voiced a belief that patients are central to all decisions (*N* = 6) and recalled examples where patient preference data has had an impact on HTA outcomes (*N* = 8): *“All healthcare should be patient-centered – the more we include patient preferences in decision-making the more relevant the decisions*,” *“Anecdotally, I have witnessed submissions where the economic case was poor, and the committee appeared reluctant to accept, only for the tone of questioning (and body language) of the committee to visibly improve upon hearing the patient perspective leading to an acceptance of the medicine under review.”*

Other responses expressed that the influence of patient preference data remains unclear, noting it is hard to quantify its impact and may be more of a case-to-case basis (*N* = 4). For example, one individual commented that the value of patient preference data *“varies wildly according to each individual appraisal…* *”*

## Discussion

This exploratory study surveyed members of HTA bodies and affiliated organizations who are involved in technology/drug appraisals and assessments, in order to seek their perspectives on incorporating patient preference data into HTA. Patient preference data were reported to be formally considered as part of the evidence review process for HTA by 82.5 percent of the participants, and 39.4 percent reported that most of the technology/drugs appraisals and assessments in the past 12 months had submitted patient preference data. This is an encouraging finding confirming the fact that HTA bodies are willing to incorporate patient preference data in HTA decision-making as supportive evidence (2;6). Another promising advancement is the availability of organizational guidelines on the use of patient preference data, which was noted by 15 participants, representing 45.4 percent of those who had reported using preference information. However, additional efforts need to be undertaken to extend the availability of such guidelines to more HTA bodies, especially because a lack of formal guidance was reported to be a key barrier by some participants. Leveraging existing guidelines and additional ongoing initiatives (5) could help facilitate this process in the future. It is important to acknowledge that about two-thirds of our respondents were from Canada, the United Kingdom, and Australia, countries with a demonstrated interest in patient preference data (4;6).

In Australia, the Pharmaceutical Benefits Advisory Committee (PBAC) recommends patient preference methods, such as DCEs, in their guidelines as an appropriate method to capture utility weights, including non-health benefits of medicines. While patient preference data are not systematically incorporated within the Australian HTA process, there have been recorded instances where patient preference data evidence has supported PBAC’s recommendations for a new therapy (7). Furthermore, there has been a push from other Australian HTA bodies, including the Medical Services Advisory Committee, for patient preference data generation in specific areas, such as genomic testing (8) – a national co-designed preference project is currently underway to address this call for evidence (9).

In England, the National Institute of Health and Care Excellence (NICE) does not currently incorporate patient preference data in their methods and processes. However, there are three areas where the role of patient preferences studies might be suitable. NICE Advice, when companies are exploring the value proposition of their products but might not have mature data yet. During health technology evaluations, for example, when comparing two very different treatment options (operation vs. drug treatment), when there is large preference heterogeneity among patients, which would render patient testimony less representative, or when the treatment has important benefits to patients that would not be well captured within standard methods. Finally, within NICE clinical guidelines, these studies could also help identify what treatments along a clinical pathway might be preferred by different subgroups of patients. Further work is needed to better identify and test whether patient preferences studies would indeed be of benefit to the work NICE does (10).

Despite certain advancements and initiatives (11), several barriers responsible for why patient preference data are not submitted in most assessments/appraisals were reported by participants. In addition to the lack of guidelines, time, resource, and cost constraints were the most often cited barriers. Lack of expertise and the unclear impact of patient preference data were also highlighted. Furthermore, the respondents reported that patient preference data only have a moderate level of influence on the deliberative process and the outcome of the decision in their organizations. Guidance to help increase the usefulness and impact of patient preference studies in decision-making (5) are thus important, particularly as most respondents believe that these barriers can be overcome in the next 2–3 years within their organizations.

However, this study also uncovered a potential mismatch between resources and responsibilities. Most participants (82 percent) believed patient advocacy groups should be responsible for generating and submitting patient preference data to HTA; however, these organizations are typically the most resource-strained and, as acknowledged by survey participants, patient preference data require considerable costs and resources. Literature has identified additional barriers to the use of patient preference data in HTA, such as methodological barriers concerning the validity and reliability of preference methods and procedural or normative barriers (12;13).

There are some limitations to this study. First, as previously mentioned, about two-thirds of respondents were from three English-speaking high-income countries (with substantial interest and experience in patient preference data), which therefore limits the generalizability of our findings to the broader HTA community. Moreover, our sample is limited in size, and some participants were not based in HTA agencies. In the recruitment materials, the survey called for the views of current members of HTA bodies and affiliated organizations who are involved in technology/drug appraisals and assessments, but their involvement was self-reported and could not be verified. Future research should investigate the views of a greater number of HTA bodies, especially those who currently demonstrate less use of patient preference data. In addition, a survey provides no deep assessment of the subject and could be considered an explorative step. More in-depth qualitative research could be of interest to better understand how the use of patient preference data in HTA could be facilitated or improved. In particular, based on this survey, the HTAi/PCIG/PPPS group is conducting a second phase of research consisting of semi-structured collaborative workshops with members of HTA bodies and affiliated organizations. This research aims to gain more in-depth insights on participants’ views and is being run in partnership with patient representatives and researchers who specialize in patient preference methods, to help facilitate discussion between different stakeholders.

## Conclusion

In line with the increasing interest in patient preference data, this explorative study revealed that most surveyed HTA bodies and affiliated organizations already use patient preference data, to some extent, in their evidence review process, and they see a clear role and value of patient preference data. However, to more broadly and meaningfully incorporate patient preference data as a standard part of the HTA decision-making process, certain barriers need to be addressed. These include concerns about time, resources, and cost constraints (which have implications for which groups are able to generate patient preference data), as well as a lack of formal guidelines in some countries and a general lack of clarity on the impact of patient preference data in HTA. Encouragingly, participants believe most obstacles can be overcome, paving the way for a second phase of research involving in-depth collaborative workshops with HTA representatives.

## Supporting information

Hiligsmann et al. supplementary material Hiligsmann et al. supplementary material

## Supplementary material

The supplementary material for this article can be found at https://doi.org/10.1017/S0266462324000138.
