# Supplementary material for: HTA community perspectives on the use of patient preference information: lessons learned from a survey with members of HTA bodies
Source: Int J Technol Assess Health Care. 2024 Mar 5;40(1):e17. doi: 10.1017/S0266462324000138 (PMC11569952; doi:10.1017/S0266462324000138)
Supplement: Hiligsmann et al. supplementary material [file S0266462324000138sup001.docx]

Appendix Table 1. Description of sample countries and organisations (n=40)

|  | Number |
| --- | --- |
| **Canada**  Institut national D’excellence en santé et services sociaux (INESSS)  British Columbia Health Technology Assessment Office (BC HTAC)  Canadian Agency for Drugs and Technologies in Health (CADTH)  Ontario Genetics Advisory Committee  Public Health Ontario  University of Calgary | **11**  4  2  2  1  1  1 |
| **United Kingdom**  [The National Institute for Health and Care Excellence](https://www.nice.org.uk/)  University of Glasgow  Health Technology Wales  Healthcare improvement Scotland (SMC)  Scottish Health Technology Group  Scottish Medicines Consortium | **11**  5  2  1  1  1  1 |
| **Australia**  Adelaide Health Technology Assessment (AHTA)  The Australian National University (ANU)  Medical Services Advisory Committee (MSAC) | **4**  2  1  1 |
| **Taiwan**  Center for Drug Evaluation – HTA Division (CDE/HTA) | **3**  3 |
| **USA**  Institute for Clinical and Economic Review (ICER) | **3**  3 |
| **The Netherlands**  National Health Care Institute  Radboudumc University Medical Center | **2**  1  1 |
| **South Africa**  Health Economics and Epidemiology Research Office (HE^2^RO) | **2**  2 |
| **Germany**  Institut für Qualität und Wirtschaftlichkeit im Gesundheitswesen (IQWIG) | **1**  1 |
| **Greece**  University of Peloponnese | **1**  1 |
| **Poland**  Agency of Health Technology Assessment and Tariff System | **1**  1 |
| **Spain**  Basque Office for Health Technology Assessment (OSTEBA-HTA) | **1**  1 |
